# Dexamethasone does not diminish sugammadex reversal of neuromuscular block – clinical study in surgical patients undergoing general anesthesia

**DOI:** 10.1186/s12871-016-0254-6

**Published:** 2016-10-21

**Authors:** Katja Rezonja, Tomaz Mars, Ales Jerin, Gordana Kozelj, Neva Pozar-Lukanovic, Maja Sostaric

**Affiliations:** 1 Department of Anaesthesiology and Intensive Therapy, University Medical Centre Ljubljana, Zaloška 7, Ljubljana, 1000 Slovenia; 2 Institute of Pathophysiology, Faculty of Medicine, University of Ljubljana, Ljubljana, Slovenia; 3 Institute of Clinical Chemistry and Biochemistry, University Medical Centre Ljubljana, Ljubljana, Slovenia; 4 Institute of Forensic Medicine, Faculty of Medicine, University of Ljubljana, Ljubljana, Slovenia

**Keywords:** Rocuronium, Sugammadex, Dexamethasone, Neuromuscular block

## Abstract

**Background:**

Sugammadex reverses neuromuscular block (NMB) through binding aminosteroid neuromuscular blocking agents. Although sugammadex appears to be highly selective, it can interact with other drugs, like corticosteroids. A prospective single-blinded randomized clinical trial was designed to explore the significance of interactions between dexamethasone and sugammadex.

**Methods:**

Sixty-five patients who were anesthetized for elective abdominal or urological surgery were included. NMB was assessed using train-of-four stimulation (TOF), with rocuronium used to maintain the desired NMB depth. NMB reversal at the end of anaesthesia was achieved using sugammadex. According to their received antiemetics, the patients were randomized to either the granisetron or dexamethasone group. Blood samples were taken before and after NMB reversal, for plasma dexamethasone and rocuronium determination. Primary endpoint was time from sugammadex administration to NMB reversal. Secondary endpoints included the ratios of the dexamethasone and rocuronium concentrations after NMB reversal versus before sugammadex administration.

**Results:**

There were no differences for time to NMB reversal between the control (mean 121 ± 61 s) and the dexamethasone group (mean 125 ± 57 s; *P* = 0.760). Time to NMB reversal to a TOF ratio ≥0.9 was significantly longer in patients with lower TOF prior to sugammadex administration (Beta = −0.268; *P* = 0.038). The ratio between the rocuronium concentrations after NMB reversal versus before sugammadex administration was significantly affected by sugammadex dose (Beta = −0.375; *P* = 0.004), as was rocuronium dose per hour of operation (Beta = −0.366; *p* = 0.007), while it was not affected by NMB depth before administration of sugammadex (Beta = −0.089; *p* = 0.483) and dexamethasone (Beta = −0.186; *p* = 0.131). There was significant drop in plasma dexamethasone after sugammadex administration and NMB reversal (*p* < 0.001).

**Conclusions:**

Administration of dexamethasone to anesthetized patients did not delay NMB reversal by sugammadex.

**Trial registration:**

The trial was retrospectively registered with The Australian New Zealand Clinical Trials Registry (ANZCTR) on February 28th 2012 (enrollment of the first patient on February 2nd 2012) and was given a trial ID number ACTRN12612000245897 and universal trial number U1111-1128-5104.

## Background

Sugammadex is the first selective muscle-relaxant-binding agent that acts by forming a complex with aminosteroid neuromuscular blocking agents (e.g., rocuronium). This results in a rapid decrease in free rocuronium in the plasma [1], and causes a shift from the acetycholine receptors in the neuromuscular junction, down the concentration gradient into the plasma. This allows rapid and effective reversal of neuromuscular block (NMB) of any depth. Sugammadex is excreted both rapidly and virtually unchanged via the urine [1, 2], as is the rocuronium–sugammadex complex, which resembles the pharmacokinetic features of sugammadex [1, 3, 4].

Corticosteroids have wide applications in anaesthesiology, as they are one of the most commonly prescribed drugs in patients with chronic diseases [5]. Furthermore, corticosteroids have an important role in states of hyper-reactive airway [6], anaphylaxis [7, 8], septic shock [9, 10], and laryngeal [11], cerebral [12, 13], and surgical edema [14], and they are also used in conjunction with multimodal analgesia [15–17] and for the prevention of postoperative nausea and vomiting [18–20]. Among the corticosteroids, dexamethasone is the most commonly used for the treatment of oedema and analgesia, and for the prevention of postoperative nausea and vomiting. Structurally, dexamethasone closely resembles the aminosteroid neuromuscular blocking agents, and therefore concerns have been raised about possible interference of such corticosteroids in the action of sugammadex [21–24].

There are two types of possible interactions of drugs with sugammadex that need to be taken into consideration: capturing and displacement. Capturing interactions can occur with oral contraceptives, where their free and active concentrations can be reduced by sugammadex coadministration to such an extent that it has the effect of missing a daily dose of contraceptives [25, 26]. Displacement potential was tested in early in vitro studies by Zhang [25], where isothermal titration calorimetry showed that over 40 lipophilic, steroid, and non-steroid drugs have some potential for interactions with sugammadex, although these affinities were 120-fold to 700-fold lower than that for the aminosteroid neuromuscular blocking agent rocuronium. The same method was used in the study of Zwiers et al. [26], where 300 drugs were tested and modeled to determine possibile drug interacts with sugammadex. Of all of these tested compounds, only three were considered possible for the displacement of rocuronium from sugammadex: toremifene, fusidic acid and flucloxacillin [26, 27]. According to these model-based chemical studies and the theoretical molecular features, sugammadex appears highly selective for aminosteroid neuromuscular blocking agents, with the minimal possibility of interactions with other drugs.

This study was designed to investigate the in vivo significance of previously observed in vitro interactions between dexamethasone and sugammadex [21, 22] in surgical patients undergoing general anaesthesia, where sugammadex was used to reverse rocuronium-induced NMB. To explore this potential interaction we hypothesized that less sugammadex is available for rocuronium binding. In agreement, the plasma rocuronium concentrations increases to a lesser extent after sugammadex application in patients treated with dexamethasone, in comparison to patients without dexamethasone treatment.

## Methods

This prospective, single-blinded, randomized, parallel-group, single-centre study was retrospectively registered with the Australian and New Zealand Clinical Trial Registry (ANZCTR) and was assigned trial number ACTRN12612000245897 and universal trial number U1111-1128-5104. The study protocol was approved by the National Medical Ethics Committee of the Ministry of Health of the Republic of Slovenia (permit number 161/02/11). Written informed consent was obtained from the patients enrolled. The study was performed at the Department of Anaesthesiology and Intensive Therapy, University Medical Centre Ljubljana, Slovenia, and conducted according to the Declaration of Helsinki, International Conference on Harmonization Guidelines and Good Clinical Practice.

### Subject recruitment

The patients included were aged 18 years or more, with American Society of Anaesthesiologists physical status I-III, and were anesthetized for elective abdominal or urological surgery for which they needed tracheal intubation and deep NMB throughout the procedure. The exclusion criteria were: lack of consent, diagnosed neuromuscular disease, anticipated difficult intubation, severe kidney failure, personal or family history of malignant hyperthermia, known allergic reaction to any of the anaesthetics used, pregnancy or nursing, and taking oral contraceptives or drugs already known to interact with sugammadex. The patients who complied with all of the inclusion and exclusion criteria were randomly assigned to the control (granisetron) group and the observed (dexamethasone) group. Randomization was performed using random number-generator, and the allocation sequence was concealed from the researcher enrolling and assessing participants in sequentially externally numbered, opaque and sealed envelopes.

### Study procedure

On arrival in the operating room, standard monitoring with pulse oximetry, capnography and electrocardiography was installed, followed by insertion of an intra-arterial cannula for invasive blood pressure measurement. The depth of anaesthesia was followed according to the bispectral index.

Anaesthesia was induced with propofol (1–2 mg/kg; IV) or etomidate (2 mg/kg; IV) and fentanyl (3–5 μg/kg; IV). Before administration of the neuromuscular blocking agent, their neuromuscular transmission was assessed by acceleromyography, using a TOF-Watch® SX neuromuscular transmission monitor (Organon Ireland Ltd, Merck and Co, Inc, Swords, Dublin, Ireland), at the ulnar nerve at 15 s intervals until stabilization. During the TOF-Watch® SX calibration period, a laryngeal mask was inserted and anaesthesia was maintained with inhaled sevoflurane (minimal alveolar concentration ≥1) in an air/oxygen mixture with intermittent positive-pressure ventilation of the patient’s lungs to achieve a normal end-tidal carbon dioxide (4.5–5.5 kPa). Calibration of the TOF was performed according to good clinical practice guidelines [28]. Rocuronium (0.6 mg/kg; IV) was then administered, and when an adequate depth of NMB was reached (according to TOF measurements), tracheal intubation was performed, and intermittent positive-pressure ventilation continued with sevoflurane in an air/oxygen mixture with additional boluses of fentanyl (2–3 μg/kg; IV). The NMB was further assessed throughout the procedure using repetitive TOF stimulation, and IV rocuronium 0.1–0.2 mg/kg was administered accordingly, to maintain the desired depth of NMB. This was maintained until the reversal of NMB using sugammadex (200 mg; IV), for recovery of the T4/T1 TOF ratio to ≥0.9 at the end of anaesthesia.

All of the patients received an antiemetic for prophylaxis of postoperative nausea and vomiting. Following intubation, the patients were randomized by the opening of an opaque envelope containing a computer-generated allocation to either control group (received granisetron (1 mg; IV) at the beginning of the operation) or dexamethasone group – these patients received dexamethasone (0.15 mg/kg; IV) 5 min to 10 min before the administration of sugammadex. Both prior to sugammadex administration and after NMB reversal to TOF ≥0.9, a blood sample (4 ml) was withdrawn from the intra-arterial line. Tubes without additives were used, the samples were centrifuged, and the serum frozen at −20 °C until analysis.

#### Determination of plasma rocuronium and dexamethasone concentrations

The rocuronium concentrations in the plasma samples obtained from the patients in the control and dexamethasone groups were determined at the Institute of Forensic Medicine, Faculty of Medicine, University of Ljubljana. For this purpose, a liquid chromatography–tandem mass spectrometry (LC-MS/MS) method was developed and validated according to recent recommendations for method validation in analytical toxicology [29], which were based on the International Conference on Harmonization, Harmonized Tripartite Guideline Validation of Analytical Procedures. The sugammadex–rocuronium complex dissociated on the chromatographic columns during the liquid chromatography, and therefore the data for rocuronium indicate the total (captured plus free) rocuronium in the plasma.

The plasma dexamethasone concentrations in the samples from patients in the dexamethasone group were determined at the Institute of Clinical Chemistry and Biochemistry, University Medical Centre Ljubljana. A competitive colorimetric enzyme-linked immunosorbent assay was used for the analyses (ID Laboratories Inc., London, Canada), with the limit of quantification of 50 μg/l, and a within-run variability of <10 %. All of these samples were analyzed as one batch.

### Statistical analysis

The primary study endpoint was the time from sugammadex administration to NMB reversal, measured as the recovery of muscle strength to a TOF ratio ≥0.9. The secondary endpoints were the ratios between the dexamethasone (test group) and rocuronium concentrations (both groups) after NMB reversal to a TOF ratio >0.9 versus the dexamethasone and rocuronium concentrations, respectively, prior to administration of sugammadex.

Due to the lack of available literature on blood concentrations of dexamethasone and rocuronium, sample size was determined on the basis of previous sugammadex studies [30–34] where the mean time from moderate or profound rocuronium-induced NMB to NMB reversal with a TOF ratio >0.9 was studied. We considered a 20 % increase in the time to NMB reversal to be clinically relevant. Assuming a power of 80 % for a two-sided test of difference in proportion at the 5 % significance level, and allowing for a 10 % drop-out due to protocol violation, we calculated that 33 patients per group were required.

The demographic data of the control and dexamethasone groups were compared using Students’ t-tests or Mann–Whitney U-tests for continuous variables, or *χ* ^2^ tests for nominal variables. For paired measurements (rocuronium and dexamethasone concentrations), paired t-tests or Wilcoxon signed rank tests were used.

Two models of multiple linear regression were constructed, the first to examine the relationship between dexamethasone and the time to reversal of the rocuronium-induced NMB with sugammadex, and the second to determine whether dexamethasone affects the ratio between the rocuronium concentrations after NMB reversal versus before sugammadex administration. Beside the group (control/dexamethasone) variable, two (depth of NMB before dexamethasone administration (baseline TOF, with TOF 1–4) and sugammadex dose) or three (depth of NMB before dexamethasone administration (baseline TOF, with TOF 1–4), sugammadex dose and rocuronium dose per hour of operation) independent variables were added in the first and second regression model, respectively. To meet the assumptions of the linear regression model, sugammadex dose and rocuronium dose per hour of operation were logarithmically transformed.

The effects of the time interval between the two blood samples that were withdrawn and the sugammadex dose on the ratio of dexamethasone concentrations after NMB reversal versus before sugammadex administration were assessed within the dexamethasone group using one-way analysis of variance (ANOVA). In the ANOVA model, the independent variables were categorized as follows: the patients who received 2–3 mg/kg sugammadex vs. 3–4 mg/kg sugammadex, and the patients in whom the time between withdrawal of blood samples was ≤5 min vs. >5 min.

## Results

**Fig. 1:**
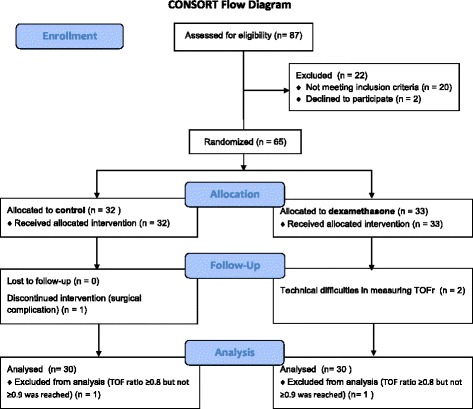
Consolidated Standards of Reporting Trials (CONSORT) diagram of study cohort. TOF – Train-of four

Based on the power analysis, 65 patients were randomized to either the control (granisetron) or dexamethasone group, of whom 62 completed the study per protocol (Fig. 1). One patient in the control group was excluded from the study due to a surgical complication, and technical difficulties when measuring the TOF were encountered with two patients from the dexamethasone group. In two patients among those who completed the study (one in the control and one in the dexamethasone group), a TOF ratio ≥0.8 but not ≥0.9 was reached, so these two patients were excluded from further analysis.

The baseline characteristics of the control and dexamethasone groups are summarized in Table 1. There were no significant differences in these parameters between the two treatment groups.

**Table 1:** Baseline characteristics for the control and dexamethasone patient groups

Patient baseline characteristic^*a*^	Control group (*n* = 31)	Dexamethasone group (*n* = 31)	*P*
Age [years (interquartile range)]	62 (52–68)	63 (52–71)	0.826
Gender: male [n (%)]	16 (51.6)	16 (51.6)	1.000
Body weight [kg (interquartile range)]	75 (70–88)	74 (63–85)	0.301
American Society of Anesthesiologists physical status (interquartile range)	2 (2–3)	2 (2–3)	0.836
Surgery duration (intubation-extubation time) [h (±standard deviation)]	2.42 (±0.83)	2.35 (±0.54)	0.790
Rocuronium dose per hour [mg/h (±standard deviation)]	41.83 (±12.21)	42.01 (±16.79)	0.525
Sugammadex dose [mg/kg (±standard deviation)]	2.62 (±0.48)	2.81 (±0.58)	0.291
Depth of NMB^*b*^ before sugammadex administration [TOF^*c*^ (interquartile range)]	0 (0–2)	0 (0–1)	0.070

^a^Data are medians (interquartile range) or means (±standard deviation)

^b^ *NMB* neuromuscular block

^c^ *TOF* train-of-four stimulation

### Dexamethasone effect on time to reversal of the NMB by sugammadex

For the time to TOF ratio ≥0.9, there were no statistically significant differences between the control (121 ± 61 s) and the dexamethasone group (125 ± 57 s; *P* = 0.760).

The first multiple linear regression model analysed the variables predicting the time to TOF ratio ≥0.9 and included group (control or dexamethasone), depth of NMB before sugammadex administration, and sugammadex dose (logarithmically transformed). The model was statistically significant (*P* = 0.049) and explained about 10 % of the population variance of the outcome (Table 2). The depth of NMB before administration of sugammadex had a statistically significant negative weight (Beta = −0.268; *P* = 0.038), which indicated that in patients with a lower baseline TOF (before sugammadex administration) the time to NMB reversal to a TOF ratio ≥0.9 tended to be longer. The weights for sugammadex dose (Beta = −0.237; *P* = 0.069) and group (control versus dexamethasone group; Beta = −0.054; *P* = 0.676) were not statistically significantly different from zero.

**Table 2:** Summary of multiple regression analysis for variables predicting the time to TOF ratio ≥0.9 (*n* = 60)

	B	SE (B)	β	*P*-value	F (*df*)	*P*-value	Adj. R^2^
Model					2.786 (3, 56)	0.049*	0.083
Constant	69.881	39.279		0.081			
Group	−6.293	14.981	−0.054	0.676			
Baseline TOF value	−15.145	7.112	−0.268	0.038*			
Sugammadex dose^a^	72.250	38.928	0.237	0.069			

*Abbreviations*: *B* variable estimate, *SE(B)* standard error of the variable estimate, *β* standardized estimate, *F (df)* F statistics (degrees of freedom), *Adj. R* ^*2*^ adjusted proportion of the variance explained by the model, *TOF* train of four

^a^Logarithmically transformed; *p < 0.05

### The effects of dexamethasone on rocuronium concentrations ratio

**Fig. 2:**
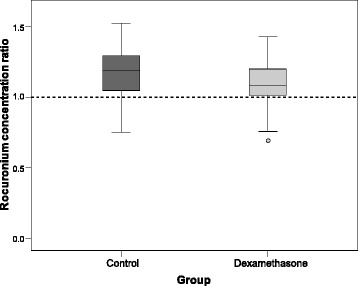
Dexamethasone effects on rocuronium concentration ratios in the control and dexamethasone groups. Box plots for the measurements of the ratio of rocuronium concentration after NMB reversal (to a TOF ratio ≥0.9) to rocuronium concentration before sugammadex administration, in patients in control and dexamethasone groups (as indicated). Data are medians with interquartile range

When comparing the ratio of the rocuronium concentrations after NMB reversal to the concentration before sugammadex administration, we did not find any statistically significant differences between the control and dexamethasone groups (control: mean 1.17 ± 0.19; dexamethasone: mean 1.09 ± 0.15; *P* = 0.090; Fig. 2).

With the second multiple linear regression model, we investigated the factors that could affect the aforementioned ratio. This model was statistically significant (*P* = 0.005) and explained about 20 % of the population variance of the outcome (Table 3). Sugammadex dose (Beta = −0.375; *P* = 0.004) and rocuronium dose per hour of operation (Beta = −0.366; *p* = 0.007) were statistically significant predictors whereas the depth of NMB before sugammadex administration (Beta = −0.089; *p* = 0.483) and addition of dexamethasone (Beta = −0.186; *p* = 0.131) did not statistically significantly predict the rocuronium concentrations ratio.

**Table 3:** Summary of multiple regression analysis for variables predicting the rocuronium concentrations ratio (*n* = 60)

	B	SE (B)	β	*P*-value	F (*df*)	*P*-value	Adj. R^2^
Model					4.242 (4, 55)	0.005*	0.180
Constant	2.303	0.339		0.000			
Group	−0.066	0.043	−0.186	0.131			
Baseline TOF value	−0.015	0.022	−0.089	0.483			
Sugammadex dose^a^	−0.345	0.115	−0.375	0.004*			
Rocuronium dose per hour of operation^a^	−0.214	0.076	−0.366	0.007*			

*Abbreviations*: *B* variable estimate, *SE(B)* standard error of the variable estimate, *β* standardized estimate, *F (df)* F statistics (degrees of freedom), *Adj. R* ^*2*^ adjusted proportion of the variance explained by the model, *TOF* train of four

^a^Logarithmically transformed; *p < 0.05

### Analysis of factors affecting plasma dexamethasone concentration

**Fig. 3:**
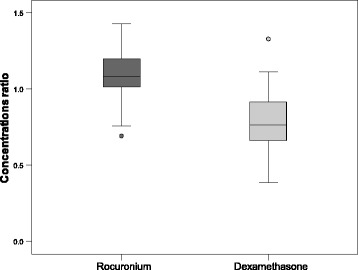
Comparison of rocuronium and dexamethasone concentration ratios in the dexamethasone group. Box plots for the measurements of the ratios of rocuronium and dexamethasone concentrations after NMB reversal (to a TOF ratio ≥0.9) to the rocuronium and dexamethasone concentrations, respectively, before sugammadex administration, in the dexamethasone group. Data are medians with interquartile range

Although there was a statistically significant difference (*p* < 0.001) between the dexamethasone concentrations before sugammadex administration (810 ± 283 μg/l) and after NMB reversal to a TOF ratio ≥0.9 (604 ± 208 μg/l, Fig. 3), ANOVA did not reveal significant effects of sugammadex dose (2–3 mg/kg (*n* = 23) vs. 3–4 mg/kg (*n* = 8); *p* = 0.729) or time between the withdrawal of blood samples (≤5 min (*n* = 12) vs. >5 min (*n* = 19); *p* = 0.524).

## Discussion

In the present study, we have shown that previously observed in vitro interactions between sugammadex and dexamethasone [21, 22] are not reflected in the recovery from rocuronium-induced NMB by sugammadex in anesthetized patients.

We first quantified the possible binding of dexamethasone to sugammadex by measuring the time to TOF ratio ≥0.9. Our results show, that the time to TOF ratio ≥0.9 is not delayed by the administration of dexamethasone before sugammadex reversal of rocuronium-induced NMB, which is in accordance with recent clinical studies by Buonanno et al. [24] and Gulec et al. [23]. In both of these studies dexamethasone was used for prevention of postoperative nausea and vomiting. Buonanno et al. retrospectively compared the effect of 8 mg dexamethasone at the induction of anesthesia or just before sugammadex adminstration on reversal time in adults [24] while Gulec et al. prospecitvely studied the reversal time of sugammadex on rocuronium induced NMB in children who received 0.5 mg/kg of dexamethasone at the induction of anesthesia [23]. In comparison to these studies, we used low-dose fixed dexamethasone concentration (0.15 mg/kg) just prior to sugammadex administration. This dosing of dexamethasone was chosen based on previous studies where it effectively reduced the incidence of postoperative nausea and vomiting [35–37], while the timing of its administration was potentially less appropriate, as we (like Buonanno et al. [24]) endeavoured to achieve higher dexamethasone concentrations in the serum of patients. It was thus not possible to assess the incidence of postoperative nausea and vomiting, which might have provided some further insight into the second type of interaction that is possible with sugammadex, i.e., capturing. Should one be able to eliminate the time factor in the dexamethasone administration, it would be of interest to evaluate these results in the context of our previous study, where sugammadex attenuated dexamethasone effects on functional innervation and on constitutive interleukin-6 (IL-6) secretion in in vitro co-cultures of human muscle cells and rat spinal cord explants [21].

We then further evaluated possible interaction between dexamethasone and sugammadex by determining plasma dexamethasone (dexamethasone group) and rocuronium concentrations (both groups) before sugammadex administration and after NMB reversal.

We observed a drop in dexamethasone concentration, which could be explained by its binding by sugammadex; however, as the NMB reversal was not delayed compared to the control group, and furthermore, the sugammadex dose did not affect the magnitude of the dexamethasone drop, we concluded it is more plausible the dexamethasone was bound by plasma proteins (especially albumin) and underwent a re-distribution to peripheral compartments, rather than being bound solely by sugammadex.

In order to indirectly detect the interactions between dexamethasone and sugammadex, we measured the plasma rocuronium concentrations, which increased after sugammadex administration. This observation is in agreement with previous studies [38–40] and can indeed be explained by rapid rocuronium encapsulation by sugammadex, which would create a concentration gradient that favours the movement of the remaining free rocuronium away from the neuromuscular junction and into the plasma. We observed that in comparison to the control group, in the patients who received dexamethasone, the rise in rocuronium concentrations was smaller, although this difference did not reach statistical significance. Therefore, our hypothesis (i.e., that because of the interaction between dexamethasone and sugammadex, less sugammadex is available for binding to rocuronium, and consequently the plasma rocuronium concentrations would increase to a lesser extent compared to the control group) was not confirmed.

Based on this clinical evaluation of NMB reversal by sugammadex and the laboratory determinations of the dexamethasone and rocuronium concentrations, we could not translate the results from our in vitro studies into clinical practice. In co-cultures of human muscle cells innervated with rat embryonic spinal cord explants, sugammadex diminished dexamethasone effects on constitutive IL-6 secretion [21], and high dexamethasone concentrations attenuated the reversal of rocuronium-induced NMB by sugammadex [22]. We can therefore assume that in human plasma, the binding between dexamethasone and sugammadex does not promote clinically significant consequences in terms of NMB reversal delay. There could be at least two possible explanations for this.

First, the plasma dexamethasone concentrations determined in in vivo after low-dose dexamethasone administration (0.15 mg/kg) are lower than those used in previous in vitro studies [21, 22]. The mean plasma concentration of dexamethasone (before administration of sugammadex) was approximately five times lower than dexamethasone concentration that resulted in the peak in vitro effect (10 μM). The latter had reportedly been reached only after high-dose dexamethasone treatment, which can be used during cardiac surgery [22, 41–45]. Hence, the sufficiently high plasma dexamethasone concentration might be critical for its complexion with sugammadex.

Second, under in vivo conditions, there are several physiological factors that can affect the binding between dexamethasone and sugammadex that cannot be predicted by in vitro conditions. This can be substantially explained by looking into the pharmacokinetics of the drugs involved. First, the greater portion of intravenous dexamethasone, 75 ± 4 %, is bound mainly by albumin [46] and the remainder, the unbound dexamethasone is theoretically suitable for interaction with sugammadex. Furthermore, the apparent volume of distribution for dexamethasone (normalized for 70 kg bodyweight) would be 65.7 ± 17.3 l [47], such that expected serum concentration for 70 kg person (receiving altogether 10.5 mg dexamethasone) would be 160 ± 61 μg/l (0.4 ± 0.2 μM), which is considerably lower than our measured mean concentration just before the reversal of NMB (810 μg/l ± 283 μg/l, which corresponds to 2.1 μM ± 0.7 μM) and mean concentration after NMB reversal to TOF ratio ≥0.9 by sugammadex (604 ± 208 μg/l, which corresponds to 1.8 ± 0.6 μM). We therefore assume that both blood samples were drawn before steady state was reached and our aim – to achieve high plasma level of dexamethasone – was therefore accomplished. This allows us to speculate about the impact of higher dexamethasone doses (that could conceivably be used in certain clinical conditions and that have also been used in in vitro studies [21, 22]) on sugammadex reversal of NMB. To elaborate even further we can take into account the pharmacokinetic profile of sugammadex, which has a volume of distribution similar to extracellular fluid (11–14 l) and it does not bind to plasma proteins [48]. The sugammadex dose of 200 mg for a 70 kg bodyweight theoretically results in the plasma concentration of 18182 – 14285 μg/l (8.3–6.6 μM). Unfortunately, sugammadex plasma concentration was not determined in our study and one can only speculate whether these theoretical steady-state concentrations reflect the actual plasma concentration, especially 2 min after administration. Furthermore, as previous studies have demonstrated, 2 min after administration of 2 mg/kg sugammadex the total (rocuronium-bound and free) plasma concentrations of sugammadex was about 20000–30000 μg/l (13.8–9.2 μM) [40, 49], which is also higher than theoretical steady-state concentrations. Considering these calculations, and further assuming all dexamethasone molecules would be captured by sugammadex immediately after its administration, about 77–85 % of surplus sugammadex would still remain available for rocuronium binding (expected steady-state concentration for a 70 kg bodyweight after receiving 0.6 mg rocuronium (mean volume of distribution of 14.2 l [50]) would be 2957 μg/l, which corresponds to 5.6 μM). Finally, besides the high protein binding properties dexamethasone lacks the charged quaternary nitrogen on the ammonium group of neuromuscular blocking agents that binds to the carboxyethyl side chains of sugammadex [51]. This explains the aforementioned low association rate constant between dexamethasone and sugammadex (less than 1000 mol/l) in contrast to high rocuronium-sugammadex association rate constant (1.79 × 10^7^ mol/l) [26] and further supports assumption that clinically important NMB delay due to dexamethasone would be highly unlikely.

Nevertheless, we cannot extrapolate our findings on corticosteroids in general. Namely, study by Zwiers et al. has revealed that dexamethasone shows the lowest displacement potential among corticosteroids [26], so based on the present and previous studies [21–24] the clinically relevant interactions between sugammadex and other corticosteroids cannot be excluded and further studies are required.

When interpreting our findings, one should take into consideration the limitations of the study. As already mentioned, sample size was determined on the basis of previous sugammadex studies [30–34] instead of conducting a preliminary study. Next, the sugammadex dose was not adjusted to depth of NMB (TOF count), but was rather generalized to all patients (one vial, 200 mg), which was then later calculated to mg/kg sugammadex for statistical analysis. To compensate for this, the data were analyzed using a regression model to adjust for different sugammadex concentrations. Furthermore, enrolment of another group of patients in whom NMB reversal would be promoted by acetylcholinesterase inhibitors (and not by sugammadex) would have helped us distinguish between the decrement in dexamethasone concentration due to its re-distribution to peripheral compartments and to the decrement attributable to its complexing with sugammadex.

Regarding the choice of the TOF ratio ≥0.9 threshold, it should be noted that we could have analyzed complete data without excluding the two patients who had not reached the threshold. The simplest approach would be to assign the maximum observed time (optionally increased by a small random value) to those two patients. A more valid approach would be to analyze the data as censored, e.g., using a Cox regression model. We tried both approaches and obtained essentially identical results to those reported (in terms of statistical significance of the model as a whole as well as of the individual predictors). The effect of omitting the two patients was therefore negligible, so we opted for using the established threshold and applying a simpler data-analysis method.

## Conclusions

To conclude, the addition of dexamethasone in anesthetized patients did not delay NMB reversal by sugammadex. Although we observed a drop in plasma dexamethasone and a rise in plasma rocuronium concentrations after sugammadex administration, this did not appear to be a direct consequence of the displacement interaction between dexamethasone and sugammadex. Therefore, the significant interaction between dexamethasone and sugammadex that has been previously demonstrated in in vitro biological models was not confirmed in anesthetized patients in vivo, probably due to several intrinsic factors that influence pharmacodynamics and pharmacokinetics of both dexamethasone and rocuronium. The interaction is therefore limited to such extent that is clinically irrelevant.

## Abbreviations

ANOVA: Analysis of variance
ANZCTR: Australian and New Zealand Clinical Trial Registry
ASA: American Society of Anaesthesiologists
IL-6: Interlekukin 6
LC-MS/MS: Liquid chromatography–tandem mass spectrometry
NMB: Neuromuscular block
TOF: Train-of-four
TOFr: Train-of four ratio
